# Screening and Molecular Identification of Pectinase Producing Microbes from Coffee Pulp

**DOI:** 10.1155/2018/2961767

**Published:** 2018-04-03

**Authors:** Oliyad Jeilu Oumer, Dawit Abate

**Affiliations:** ^1^Department of Biology, Ambo University, P.O. Box 19, Ambo, Ethiopia; ^2^College of Natural Science, Addis Ababa University, P.O. Box 1176, Addis Ababa, Ethiopia

## Abstract

Application of enzymes in biotechnological process has expanded considerably in recent years. In food and related industry, major importance was being attached to the use of enzymes in upgrading quality, increasing yields of extractive processes, product stabilization, and improvement of flavor and byproduct utilization. Pectinases or pectinolytic enzymes are today one of the upcoming enzymes of the commercial sector. It has been reported that microbial pectinases account for 25% of the global food enzymes sales. For this reason, this study was undertaken with aims of screening microorganisms for the pectinase activity from coffee pulp samples and molecular identification of the potential pectinolytic isolates. In the present investigation, in total, ninety-five (95) isolates were identified from thirty coffee pulp samples. Based on characterization on the selective growth media, the isolates were grouped as actinomycete (21.06%), bacteria (65.26%), and fungi (13.68%). Among these, 31.58% showed colonies surrounded by clear zones which indicate the presence of pectinase activity. After rigorous screening steps, the isolates with high potential pectinase activity were identified molecularly by sequencing 16S rDNA region of the isolates. Based on the molecular identifications, about 70% of the isolates are under genus* Bacillus*.

## 1. Introduction

Enzymes are natural catalysts. They are produced by living organisms to increase the rate of an immense and diverse set of chemical reactions required for life. They are involved in all processes essential for life such as DNA replication and transcription, protein synthesis, metabolism, and signal transduction. Their ability to perform very specific chemical transformation has made them increasingly useful in industrial processes [1].

In nature, microorganisms have been endowed with vast potentials. They produce an array of enzymes, which have been exploited commercially over the years. Today's enzyme technology mostly depends on microbes like bacteria and actinomycetes. Potential microorganisms are highly susceptible to genetic manipulations and hence provide ample scope for strain improvement and for further investigation. Ecofriendly biotechnological processes seem to be very important as far as the modern society is concerned for which microbial enzymes are recognized as efficient tools. Thus we attempted a study to screen and report enzymes producing microbes [2].

The biotechnological potential of pectinolytic enzymes from microorganisms has drawn a great deal of attention from various researchers worldwide as likely biological catalysts in a variety of industrial processes. Pectinolytic enzymes can be applied in various industrial sectors wherever the degradation of pectin is required for a particular process. Several microorganisms have been used to produce different types of pectinolytic enzymes [3]. Microbial pectinases account for 25% of the global food and industrial enzyme sales [4, 5] and their market is increasing day by day. These are used extensively for fruit juice clarification, juice extraction, manufacture of pectin free starch, refinement of vegetable fibers, degumming of natural fibers, wastewater treatment, and cocoa and tobacco and as an analytical tool in the assessment of plant products [6, 7]. Pectinase treatment accelerates tea fermentation and also destroys the foam forming property of instant tea powders by destroying pectins. They are also used in coffee fermentation to remove mucilaginous coat from coffee beans [8, 9].

Although use of pectinases in food-processing industries has been fairly well established, the modes of action and experimental utility of several pectin-degrading enzymes have not been explored for applications in human nutrition and health. Prebiotics preferred pectic oligosaccharides during fermentation. Their abilities to protect colonocytes against* Escherichia coli* verocytotoxins, to stimulate apoptosis in human colonic adenocarcinoma, and to increase* Bifidobacteria *and* Eubacterium rectale* numbers with the subsequent increase in butyrate concentrations have also been reported [19].

Almost all the commercial preparations of pectinases are produced from fungal sources.* Aspergillus niger* is the most commonly used fungal species for industrial production of pectinolytic enzymes. Thus, owing to the enormous potential of pectinase in various sectors of industries whenever degradation of pectin is needed, it is important to undertake research in screening of microorganisms for pectinase production.

Therefore, the present study was conducted with the aims of screening pectinolytic microorganisms from coffee pulp and identifying molecularly using 16S rRNA.

## 2. Material and Methods

### 2.1. Sample Collection

A total of 30 coffee pulp samples were collected from coffee cherry processing site in Gomma, Jima zone, Ethiopia. The sample collected area is located in Oromia National Regional State of Ethiopia. It is located 390 km south west of Addis Ababa, which is the capital city of Ethiopia. The altitude of this area ranges from 1,380 to 1,680 meters above sea level; however, some points along the southern and western boundaries have altitudes ranging from 2229 to 2870 meters.

About 100 g of each sample was collected aseptically using UV-rays sterilized polythene bags. The sample containing bags were sealed and stored into 4°C refrigerator in Mycology Laboratory, Addis Ababa University, until the time of the analysis.

### 2.2. Serial Dilution

Aseptically, 10 g of Coffee Husk from each sample was pooled out and homogenized in sterile 90 ml distilled water. The homogenized samples were agitated for 1 hour at 120 rpm on INFORS HT Ecotron incubator shaker and then serially diluted until dilutions 10^−4^ and 10^−5^.

### 2.3. Media Preparation

*Starch casein agar media* were prepared by dissolving 10 g soluble starch, 0.3 g casein, 2 g KNO_3_, 2 g NaCl, 2 g K_2_HP0_4_, 0.05 g MgSO_4_·7H_2_O, 0.02 g CaCO_3_, 0.01 g FeSO_4_·7H_2_O, and 15 g agar in 1000 ml of distilled water inside 2 L Erlenmeyer flask. The pH of the medium was adjusted to 7.0 ± 0.5 using digital pH meter (OAKTON-pH110). The media were sterilized at a temperature of 121°C for 15 minutes. Finally, about 20–25 ml of the sterilized starch casein agar media was poured on sterile Petri plates in the microbiological hood and allowed to solidify at room temperature.

*Nutrient agar media* were prepared by dissolving 28 g of nutrient agar in 1000 ml of distilled water inside 2 L Erlenmeyer flask. The pH of the medium was adjusted to 7.0 ± 0.5 using digital pH meter (OAKTON-pH110). The media were sterilized at a temperature of 121°C for 15 minutes. Finally, about 20–25 ml of the sterilized nutrient agar media was poured on sterile Petri plates in the microbiological hood and allowed to solidify at room temperature.

*Malt extract agar media* were prepared by dissolving 50 g of malt extract agar powder in 1000 ml of distilled water inside 2 L Erlenmeyer flask. The pH of the medium was adjusted to 7.0 ± 0.5 using digital pH meter (OAKTON-pH110). The media were sterilized at a temperature of 121°C for 15 minutes. After sterilization, the medium allowed to cool up to 50°C and supplemented with 1 *μ*g/mL of* Chloramphenicol* to eliminate the growth of microorganisms other than fungus. Finally, about 20–25 ml of the sterilized malt extract agar media was poured on sterile Petri plates in the microbiological hood and allowed to solidify at room temperature.

### 2.4. Isolation of Actinomycetes

For the isolation of actinomycetes, 0.1 ml aliquots of samples from appropriate dilutions were inoculated onto sterilized and solidified starch casein agar medium using spread plate method (SCAM). Inoculated plates were incubated aerobically at 30°C for 3 days−2 weeks in the INFORS HT Ecotron incubator.

### 2.5. Isolation of Bacteria

For the isolation of bacteria, 0.1 ml aliquots of samples from appropriate dilutions were inoculated onto sterilized and solidified nutrient agar medium by spread plate method. Inoculated plates were incubated aerobically at 30°C for 24–48 hours in the INFORS HT Ecotron incubator.

### 2.6. Isolation of Fungi

For the isolation of fungi, 0.1 ml aliquots of samples from appropriate dilutions were inoculated on sterilized and solidified malt extract agar medium by spread plate method. Inoculated plates were incubated at 30°C for 3–8 days in the INFORS HT Ecotron incubator.

### 2.7. Purification and Preservation of Cultures

Different colonies were randomly picked from countable plates (SCAM for actinomycetes, nutrient agar for bacteria and malt extract agar for fungi) and purified by repeated streaking on the respective media. Pure cultures of each group of microorganisms were then streaked on slants of respective media and stored at 4°C for further study.

### 2.8. Presumptive Screening of Isolates for the Pectinase Activity

The isolates were preliminarily screened for pectinase activity using pectinase screening agar medium (PSAM). The pH of the medium was adjusted to 5.5 ± 0.5 before sterilization and then autoclaved with a temperature of 121°C for 15 minutes. Finally, 20–25 ml of media was poured on sterile Petri dishes in the microbiological hood and allowed to solidify at room temperature. All isolates were streaked into this media and incubated at 30°C for 24 hours to 2 weeks. At the end of the incubation period, the plates were flooded with 50 mM Potassium iodide-iodine solution. A clear halo zone around the colonies indicates the ability of an isolate to produce pectinase [20].

### 2.9. Primary Screening of Efficient Pectinase Producing Isolates

All the pectinase positive isolates were screened by inoculating them into the above-mentioned screening media. Using a flamed and cooled cork borer, a disc of actively growing pectinase positive isolate was taken and transferred to the center of screening media and then incubated at 30°C for 24 hours to 2 weeks. The ratio of the clear zone diameter to colony diameter during that span of time was measured in order to select isolates with highest pectinase activity. The largest ratio is assumed to contain the highest activity. Those isolates with highest ratio were selected for further screening.

### 2.10. Secondary Screening of Efficient Pectinase Producing Isolates

Isolates with highest clear zone diameter to colony diameter ratio in the pectin-agar plates were subjected to submerged fermentation using YEP medium. The pH of the medium, YEP, was adjusted to 7.0 ± 0.5 before sterilization and then autoclaved with a temperature of 121°C for 15 minutes. A volume of 50 mL YEP medium in 250 mL Erlenmeyer flask was inoculated with 1% inoculum. The inoculated flasks were incubated at 30°C on an INFORS HT Ecotron incubator shaker at 120 rpm. Samples from inoculated flasks were collected at regular intervals of 24 h and centrifuged at 10,000 rpm for 5 min at 4°C. The supernatant was used for measuring the enzyme activity. The enzyme activity was assayed using sodium acetate buffer, pH 6.5.

### 2.11. Pectinase Enzyme Assay

Pectinase enzyme assay was based on the determination of reducing sugars produced as a result of enzymatic hydrolysis of pectin by dinitrosalicylic acid reagent (DNS) method (Miller, 1959). For enzyme assay, 1.5 mL of freshly grown culture was taken and centrifuged at 10,000 rpm for 5 min. The supernatant (100 *μ*L) from the culture broth was served as the source of the enzyme. In addition, substrate was prepared by mixing 0.5% (w/v) citrus pectin in 0.1 M of pH 7.5 phosphate buffer.

From the prepared substrate, 900 *μ*l was added to three clean labeled test tubes; one for enzyme, one for enzyme blank, and one for reagent blank. Then, 100 *μ*l of crude enzyme was added to test tube labeled as enzyme and 100 *μ*l of distilled water was added to test tube labeled as regent blank while test tube labeled as enzyme blank remained as it was. Then, the test tubes were incubated at 50°C for 10 minutes in the water bath. After incubation 2000 *μ*l of dinitrosalicylic acid reagent (DNS) was added to the all test tubes to stop the reaction. Meanwhile, into test tube labeled as enzyme blank 100 *μ*l of crude enzyme was added after the DNS. Then, all the test tubes were placed in a boiling water bath (92°C) for 10 minutes. Finally, the tubes were cooled and optical density (OD) was measured using spectrophotometer (JENWAY 6300 UV/Vis) at 540 nm. Enzyme activity was measured against enzyme blank and reagent blank. The enzyme unit was defined as the amount of enzyme that catalyzes *μ*mol of galacturonic acid per minute (*μ*mol min^−1^) under the assay conditions.

Relative activity was calculated as the percentage enzyme activity of the sample with respect to the sample for which maximum activity is obtained:

(1) $$\begin{array}{c} \text{Relative Activity}=\frac{\text{Activity of sample}\left(U\right)\times 100}{\text{Maximum enzyme activity}\left(U\right)}. \end{array}$$

### 2.12. Molecular Identification

The potential isolates which were screened and selected on the primary phase were identified using molecular techniques.

### 2.13. Genomic DNA Extraction

The potential isolates were inoculated into 50 ml of LB broth (Luria-Bertani) and incubated at 37°C for 24–48 hours. After incubation, they were centrifuged at 5000 rpm, 4°C, for 10 min, and cells were collected for genomic DNA extraction. The genomic DNA of the isolates was extracted by using the Bacterial Genomic DNA extraction kit according to the manufacturer protocol (QIAGEN, QIAamp DNA Mini Kit).

### 2.14. Quantification and Qualification of DNA by Agarose Gel Electrophoresis

The extracted DNA was subjected to agarose gel electrophoresis along with the marker DNA (DNA Ladder) to confirm the quality and quantity. About, 1 g of agarose weighted and poured into 100 ml of 1x TAE buffer to make 1% agarose gel. The solution was heated in a microwave oven until agarose gets dissolved. The agarose solution cooled down for about 5 minutes and 0.5 *μ*g/mL of ethidium bromide (EtBr) added. Then, the agarose solution poured into a gel tray with the well comb in place and it was allowed to solidify. After being solidified, the comb was removed and the gel was placed in an electrophoretic tank consisting of 1x TBE buffer. About 3 *μ*l of the extracted DNA was mixed with 2 *μ*l of the gel loading dye (Bromophenol blue) and it was loaded into the agarose gel wells. The gel was then electrophoresed at 100 volts for about 45 minutes and it was observed in a gel documentation system.

### 2.15. PCR Amplification of the 16S rRNA Gene

PCR is used to amplify the DNA sequence in between two known sequences. The amplification of 16s rRNA gene of the isolates was carried out using PCR machine. Two specific primers, forward primer 27F (5′-(AGA GTT TGA TCM TGG CTC AG)-3′) and reverse primer 1492R (5′-(CGG TTA CCT TGT TAC GAC TT)-3′) which are complementary to the known 16S rDNA sequences, were used.

### 2.16. Setting Up the PCR Reaction and PCR Machine

Into the AccuPower® Taq PCR PreMix (10 *μ*l the master mix containing 10x* Taq *buffer, 10 mM dNTPs, 25 mM of MgCl**2**, 1 U of* Taq *DNA polymerase), 1.5 *μ*l of forward primer (27F), 1.5 *μ*l of Reverse primer (1492R), 2 *μ*l of Genomic DNA, and 5 *μ*l of PCR grade water were added and the PCR amplification was done. The thermal cycler was programmed for 35 cycles as follows:* (1) initial denaturation*, 94°C for 2 minutes;* (2) amplification*, 94°C for 45 seconds (denaturation), 56°C for 1 minute (annealing), and 72°C for 1 minute and 30 seconds (extension);* (3) final extension*, 72°C for 5 minutes and then held at 4°C until needed.

### 2.17. Quantification and Qualification of Amplified DNA Samples by Nanodrop

The amplified DNA might contain impurities of RNA and protein that can interfere in the DNA tests. About 10 *μ*l of isolated DNA sample was placed into the Nanodrop instrument and quantification and qualification of amplified DNA was done.

### 2.18. Gene Sequencing and Analysis

The PCR sequence products were purified and sequenced. The obtained sequence data were compared with known sequences in the GenBank using the basic local alignment search tool of the national center for biotechnology information (NCBI). Species were identified based on the percentage similarity with the known species sequences in the data base.

## 3. Results

### 3.1. Isolation of Microorganisms from Coffee Pulp

To isolate microorganisms from the collected coffee pulp samples, serial dilution, pour plating, and streak plating isolation techniques were used. Subsequently, the isolates were subcultured into their respective selective growth media until pure cultures were isolated. In total, ninety-five (95) isolates were identified from thirty coffee pulp samples. Based on characterization on the selective growth media, the isolates were grouped as actinomycete (21.06%), bacteria (65.26%), and fungi (13.68%). For identification purpose, the isolates were designated by prefix “Btk” and followed by their isolate numbers.

### 3.2. Screening Isolates for Pectinase Production

#### 3.2.1. Presumptive Screening

Subsequent to isolation and purification, the isolates were assessed for pectinase activity using pectinase screening agar medium (PSAM). Among the Ninety-five isolates, 31.58% showed colonies surrounded by clear zones which indicate the presence of pectinase activity.

#### 3.2.2. Primary Screening

To identify isolates with higher pectinase activity, the ratio between clear zone diameter and colony diameter was calculated. The highest ratio observed was 4.7 ± 1.2 by isolate Btk27. Those isolates which scored higher than or equal to 2.0 ± 1.5 (Mean ± SD) were considered as high enzyme producers, which accounts for 33.3% of the pectinase positive isolates (Table 1). These isolates were selected for molecular identification and further screening.

#### 3.2.3. Secondary Screening

In order to further screen the selected isolates from primary screening, submerged fermentation test was performed. Accordingly, subsequent to incubation in YEP media, aliquot samples were taken and assayed for their pectinase activity at 6.5 pH Sodium Acetate buffer. The highest enzyme activities, observed were 7.5 ± 1.17 and 7.5 ± 0.52 (U/ml) by isolates Btk25 and Btk27, respectively (Table 2). The lowest observed enzyme activity was 0.02 ± 0.01 (U/ml) by isolate 42 Btk71.

### 3.3. Molecular Identification of the Isolates

The amplified PCR products were purified and sequenced. The obtained sequence data were blasted in NCBI database and the likely microorganisms (with lower* E* value, higher identity percentage and maximum total score) were identified (Table 3).

## 4. Discussion

In recent years, the potentials of using microorganisms as biotechnological sources of industrially relevant enzymes have stimulated interest in the exploration of extracellular enzymatic activity in several microorganisms [4]. Pectinase producing microorganisms have advantage over other sources because they can be subjected to genetic and environmental manipulations to increase yield [21]. In this study, potential pectinase producing microorganisms were isolated using serial dilution, pour plating, and streak plating techniques from coffee pulp. Similarly, [22, 23] also followed similar procedures to isolate microorganisms from coffee husk and pulp. Coffee pulp is a fibrous mucilaginous material acquired during the processing of coffee cherries by wet or dry process, respectively. The presence of proteins, pectin, other sugars, and minerals and its high humidity favor the rapid growth of pectinolytic microorganisms. Moreover, the organic nature of the material makes it an ideal substrate for microbial processes for the production of value-added products [24].

Emerging new applications underline the importance of screening pectinase producing microorganisms with novel properties, greater enzyme activity, and large-scale production of these enzymes [25]. In this study, the isolates were subjected into plate agar and submerged fermentation screening methods to identify potent isolate with highest enzyme activity and enzyme activity with broad pH ranges. The study is in agreement with [26] reporting plate agar screening method used to screen native isolates for pectinase activity. Mehta et al. [27] screened bacterial strains isolated from soil and samples of vegetable using plate agar and submerged fermentation screening methods.

Pectinases are a heterogeneous group of related enzymes and according to the cleavage site; they are divided into three groups: (1) polygalacturonase; (2) pectin lyase and pectate lyase; (3) pectin esterase [28, 29]. Polygalacturonases (PGases) have been reported in many microorganisms, including* Neurospora crassa*,* Aspergillus* sp., and* Bacillus* sp. [5, 30]. Pectin esterase is reported in* Pseudomonas solanacearum*,* Aspergillus niger*,* Lactobacillus lactis*,* Penicillium occitanis*, and* A. japonicus*[4, 5, 31]. Pectate lyases are produced by many bacteria and some pathogenic fungi. They have been reported in* Erwinia carotovora*,* Pseudomonas syringae*, and* Bacillus* sp. [5, 32]. Pectin lyases have been reported to be produced by* Aspergillus japonicus*,* Penicillium* sp., and* Aspergillus *sp. [5, 30, 33]. In this study, pectinase activity is determined on the basis of measuring the amount of reducing sugar by colorimetric methods specifically using 5-dinitrosalicylate reagent method. Based on the assay procedures and characteristics of the pectinase, the pectinase of the screened and identified isolates in this study resembles polygalacturonase.

The potential isolates for pectinase production were identified molecularly using the 16S rRNA gene. Among the molecularly identified isolates about 70% of the isolates were under genus of* Bacillus*. According to Priest (1977), there was a widespread distribution of pectinolytic activity throughout the genus of* Bacillus*. Some works also had been done to produce pectinase by many strains of these genus [32, 34–37]. In addition, during screening the isolates which secrete the highest quantity of pectinase was also of genus* Bacillus*. The result is in good agreement with Namasivayam et al. [34] which reported that Bacillus* sp. *can produce large quantities of extracellular pectinase enzyme. Also, out of the ten molecularly identified isolates, six out of them have percentage similarity on the database <97%; therefore those isolates might be a novel or a new strain/species [38].

## Figures and Tables

**Table 1:** Primary screening: Isolates clear zone to colony diameter ratio.

Sr. number	Isolate	Clear zone to colony diameter ratio^*∗*^
1	Btk5	2.9 ± 1.1
2	Btk23	3.5 ± 1.8
3	Btk25	2.7 ± 0.3
4	Btk26	3.4 ± 0.7
5	Btk27	4.7 ± 1.2
6	Btk59	3.5 ± 0.8
7	Btk71	2.7 ± 1.3
8	Btk73	2.0 ± 1.5
9	Btk81	2.7 ± 0.3
10	Btk95	2.8 ± 0.9

^*∗*^Values are mean ± SD of triplicates.

**Table 2:** Secondary screening: pectinase activity (Unit/ml).

Sr. number	Strain number	Enzyme activity^*∗*^
1	Btk5	3.4 ± 1.12^b^
2	Btk23	6.6 ± 1.61^ba^
3	Btk25	7.5 ± 1.2^a^
4	Btk26	5.2 ± 0.5^ba^
5	Btk27	7.5 ± 0.5^ab^
6	Btk59	1.0 ± 0.4^b^
7	Btk71	0.02 ± 0.01^b^
8	Btk73	3.1 ± 1.9^b^
9	Btk81	0.14 ± 0.01^b^
10	Btk95	0.1 ± 0.01^b^

(i) ^*∗*^Values are mean ± S.D. of 3 replicates; (ii) Values followed by different superscripts are significantly different at (*P* < 0.05); (iii) Values followed by same superscripts are not significantly different at (*P* < 0.05).

**Table 3:** Blast (Basic Local Alignment Search Tool) table.

Isolate	Molecular identification	GenBank accession number	%similarity	Taxonomy	Habitat	Reference
Btk5	*Bacillus methylotrophicus strain EGY-SCJ5*	KC573497.1	97	*Bacillus *	Marine water sediment, Egypt	[10]

Btk23	*Bacillus pumilus strain B7 *	KF641839.1	95	*Bacillus *	Camel rumen, China	[11]

Btk25	*Bacillus subtilis strain NBT-15*	HQ244501.1	97	*Bacillus *	Penut, China	[12]

Btk26	*Bacillus amyloliquefaciens strain ASAG1*	FJ597542	95	*Bacillus *	Stored corn, China	[13]

Btk27	*Bacillus subtilis strain NBT-15 *	HQ244501.1	97	*Bacillus *	Penut, China	[12]

Btk59	*Exiguobacterium *sp.* Y11 *	EF177690.1	95	*Exiguobacterium*	Salt Mine, China	[14]

Btk71	*Pusillimonas ginsengisoli strain DCY28*	EF672089.1	96	*Pusillimonas *	Soil, South Korea	[15]

Btk73	*Bacillus methylotrophicus strain IHB B 7249*	KJ767354.1	97	*Bacillus *	*Camellia sinensis *(tea), India	[16]

Btk81	*Bacillus *sp.* SVUNM*	JX119240.1	74	*Bacillus*	Mica mines, India	[17]

Btk95	*Staphylococcus *sp.* NR7 *	EU784844.1	88	*Staphylococcus *	Sausage, China	[18]
